# Identification of novel serum autoantibodies against EID3 in non-functional pancreatic neuroendocrine tumors

**DOI:** 10.18632/oncotarget.22175

**Published:** 2017-10-31

**Authors:** Koji Hontani, Takahiro Tsuchikawa, Takaki Hiwasa, Toru Nakamura, Takashi Ueno, Toshihiro Kushibiki, Mizuna Takahashi, Kazuho Inoko, Hironobu Takano, Satoshi Takeuchi, Hirotoshi Dosaka-Akita, Masaki Kuwatani, Naoya Sakamoto, Yutaka Hatanaka, Tomoko Mitsuhashi, Hideaki Shimada, Toshiaki Shichinohe, Satoshi Hirano

**Affiliations:** ^1^ Department of Gastroenterological Surgery II, Hokkaido University Graduate School of Medicine, Sapporo 060-8638, Japan; ^2^ Department of Biochemistry and Genetics, Chiba University, Chuo Ku, Chiba 260-8670, Japan; ^3^ Department of Medical Oncology, Hokkaido University Graduate School of Medicine, Sapporo 060-8638, Japan; ^4^ Department of Gastroenterology and Hematology, Hokkaido University Graduate School of Medicine, Sapporo 060-8638, Japan; ^5^ Department of Translational Pathology, Hokkaido University Graduate School of Medicine, Sapporo 060-8638, Japan; ^6^ Department of Surgery, School of Medicine, Toho University, Ota-ku, Tokyo 143-8541, Japan

**Keywords:** pancreatic neuroendocrine tumors, SEREX, biomarker, prognostic factor, EID3

## Abstract

Pancreatic neuroendocrine tumors (pNETs) are relatively rare heterogenous tumors, comprising only 1–2% of all pancreatic neoplasms. The majority of pNETs are non-functional tumors (NF-pNETs) that do not produce hormones, and as such, do not cause any hormone-related symptoms. As a result, these tumors are often diagnosed at an advanced stage because patients do not present with specific symptoms. Although tumor markers are used to help diagnosis and predict some types of cancers, chromogranin A, a widely used tumor marker of pNETs, has significant limitations. To identify novel NF-pNET-associated antigens, we performed serological identification of antigens by recombinant cDNA expression cloning (SEREX) and identified five tumor antigens (phosphatase and tensin homolog, EP300-interacting inhibitor of differentiation 3 [EID3], EH domain-containing protein 1, galactoside-binding soluble 9, and BRCA1-associated protein). Further analysis using the AlphaLISA^®^ immunoassay to compare serum antibody levels revealed that antibody levels against the EID3 antigen was significantly higher in the patient group than in the healthy donor group (*n* = 25, both groups). In addition, higher serum anti-EID3 antibody levels in NF-pNET patients correlated with shorter disease-free survival. The AUC calculated by ROC analysis was 0.784 with moderate diagnostic accuracy. In conclusion, serum anti-EID3 antibody levels may be useful as a tumor marker for prediction of tumor recurrence in NF-pNETs.

## INTRODUCTION

Pancreatic neuroendocrine tumors (pNETs) are relatively rare tumors originating from neuroendocrine cells, which have attracted growing attention in recent years due to their increasing prevalence worldwide [1, 2]. pNETs can be classified as functional pNETs (F-pNETs) and non-functional pNETs (NF-pNETs) depending upon the presence of hormone-related symptoms. F-pNETs are often diagnosed at an early stage due to symptoms such as insulinoma related to hypoglycemia, gastrinoma, and gastrointestinal carcinoid tumors. On the other hand, patients with NF-pNETs do not present with specific symptoms, and thus, are typically diagnosed at an advanced stage [3].

Because there are few effective tumor markers for pNETs, the evaluation of anti-tumor therapy efficacy and screening of recurrence are mainly performed by imaging tests. Chromogranin A (CgA) is currently used as a tumor marker for pNETs in western populations; however, its value is influenced by various factors including comorbid conditions or medications and lack of assay standardization [4–6]. Therefore, there is a need to identify novel tumor markers that can aid with the detection and management of pNETs.

Serological identification of antigens by recombinant cDNA expression cloning (SEREX), a comprehensive method that was reported by Sahin *et al* [7] in 1995, allows the rapid and large-scale identification of tumor antigens by using patient sera as the detection platform. Two SEREX methods have been reported: one using a cDNA library constructed from tumor tissue and one using a testicular cDNA library [7–10]. In screening tumor-derived cDNA libraries, it was possible to identify tumor-associated antigens such as p53 antigen in colorectal cancer [11]. On the other hand, cancer testis antigens such as NY-ESO-1 in esophageal cancer were found using a testis cDNA library [8]. Although different libraries were used for antigen screening, both methods led to the identification of clinically relevant IgG-type tumor markers. The SEREX method has been applied to many types of cancers and has resulted in the identification of several tumor antigens including p53 antigens, and in fact, serum p53 antibodies serve as tumor markers for multiple types of cancers such as esophageal, colon, and lung cancers [11–17]. However, to the best of our knowledge, the SEREX method has not been used to identify tumor markers for pNETs, nor is there any research on autoantibodies for pNETs.

In this study, novel tumor antigens for NF-pNETs were identified using the SEREX method. Then the ability of antibodies against the identified antigens to serve as tumor markers was investigated by comparing serum antibody levels between patients and healthy donors (HDs).

## RESULTS

### Identification of NF-pNET-associated antigens by SEREX technology

**Figure 1:**
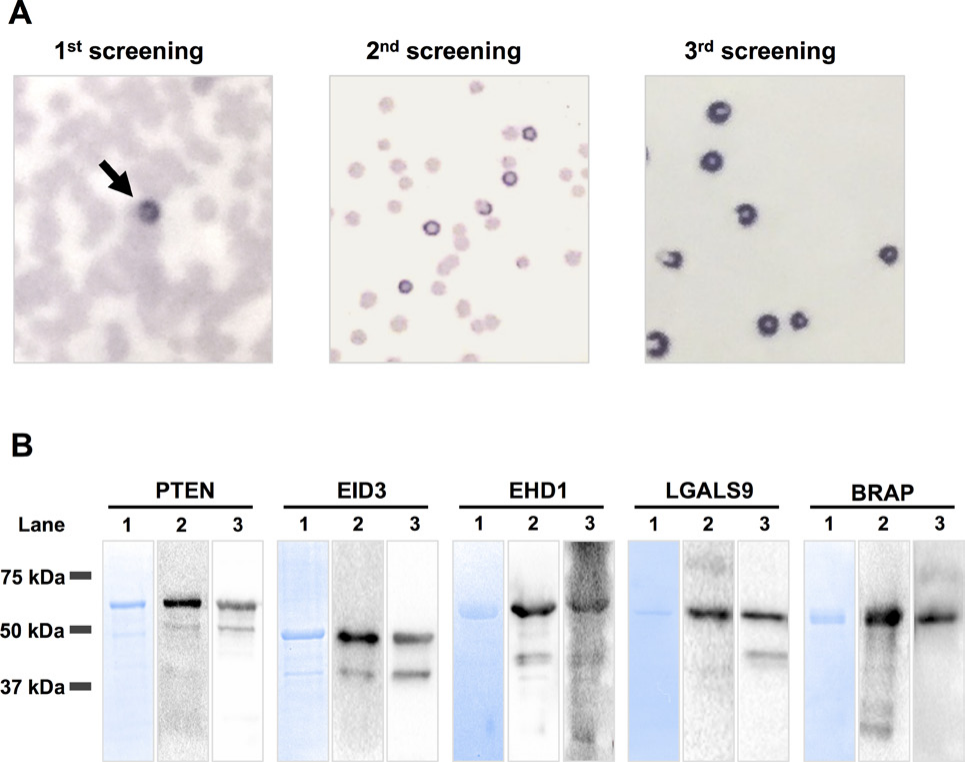
SEREX screening and western blot analysis of recombinant GST-fusion proteins (**A**) Proteins expressed from cDNA clones were transferred onto nitrocellulose membranes and screened with patient sera. The positive clone was picked up in the first SEREX screening (arrow). After the second round and third round of SEREX screening, the positive clone was monoclonalized. (**B**) Lane 1: Protein analysis of the column-purified GST-fusion protein by SDS-PAGE and Coomassie Blue staining. Lane 2: Western blotting of recombinant GST-fusion proteins using anti-GST antibody. Lane 3: Western blotting of recombinant GST-fusion proteins using antibodies against each antigen. SEREX: serological identification of antigens by recombinant cDNA expression cloning, NF-pNETs: non-functional pancreas neuroendocrine tumors.

A cDNA phage library was constructed from the mRNA of NF-pNET tumor specimens from three patients. The size of the original cDNA library was 1.6 × 10^6^ PFU/mL. A total of 2.0 × 10^7^ clones in the constructed cDNA library and testis cDNA library were screened with autologous serum from NF-pNET patients, and 20 immunoreactive clones were isolated (Figure 1A). DNA sequence analysis and the Basic Local Alignment Search Tool (BLAST) identified five distinct antigens: phosphatase and tensin homolog (PTEN), EP300-interacting inhibitor of differentiation 3 (EID3), EH domain-containing protein 1 (EHD1), galactoside-binding soluble 9 (LGALS9), and BRCA1-associated protein (BRAP) (Table 1). The region of each gene between nucleotides 1424 and 2301 in PTEN, 427 and 1487 in EID3, 738 and 2431 in EHD1, 93 and 1617 in LGASL9, and 234 and 410 in BRAP were isolated. The coding sequence encompassed nucleotides 1032–2243 in PTEN, 204–1205 in EID3, 123–1727 in EHD1, 119–1090 in LGALS9, and 205–1983 in BRAP.

**Table 1:** Genes identified by SEREX screening with serum from patients with NF-pNET

Clone name	Gene identity	NCBI Accesion Number	Library
N - 1	Phosphatase and tensin homolog (PTEN)	NM_000314	Testis
N - 2	EP300 interacting inhibitor of differentiation 3 (EID3)	NM_001008394	NF-pNET, Testis
N - 3	EH domain containing 1 (EHD1)	NM_001282444	NF-pNET
N - 4	Galectin 9 (LGALS9)	NM_002308	NF-pNET
N - 5	BRCA1 associated protein (BRAP)	NM_006768	NF-pNET

NF-pNET: non-functional pancreatic neuroendocrine tumor, NCBI: national center for biotechnology information SEREX: serological identification of antigens by recombinant expression cloning

### Confirmation of GST-fusion protein expression by western blotting

The cDNA fragments encoding the five antigens identified by SEREX screening were cloned into a pGEX vector to construct glutathione S-transferase (GST)-fusion proteins. To confirm the purification and expression of the GST-fusion proteins, they were resolved on SDS-PAGE gels. Then the proteins on the gel were either stained with Coomassie blue or were electrotransferred onto membranes for western blot analysis. Coomassie blue staining showed prominent bands at the molecular weights expected from sequence analysis (Figure 1B, lane 1). Western blotting was performed using antibodies against GST (Figure 1B, lane 2) and against each identified antigen, namely anti-PTEN, anti-EID3, anti-EHD1, and anti-LGALS9, and anti-BRAP (Figure 1B, lane 3). These results show that the GST-fusion proteins were purified and could be detected with the appropriate antibody; the smaller bands were degradation products.

### Analysis of antibody reactivity against tumor antigens using AlphaLISA^®^

**Figure 2:**
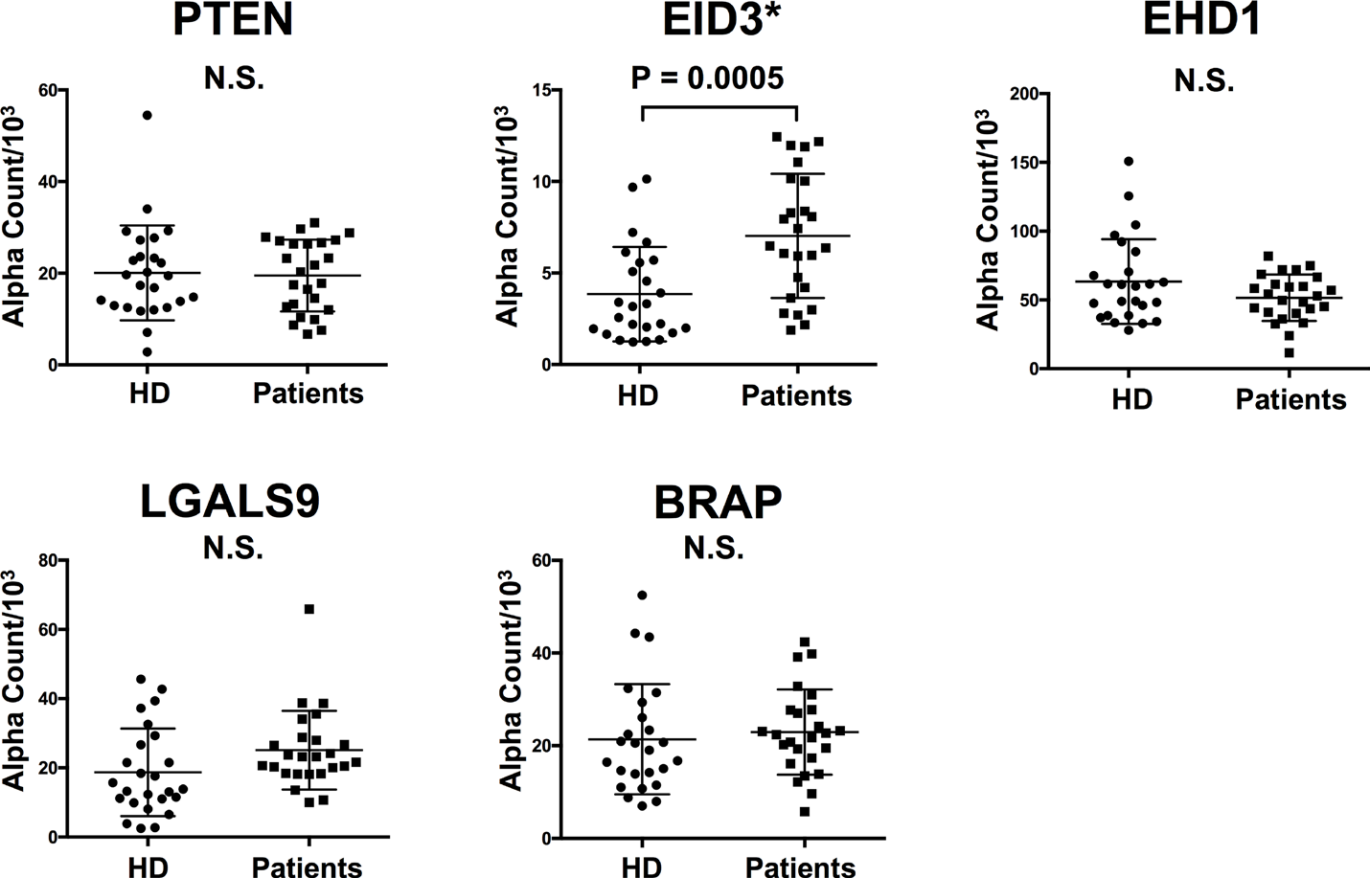
Comparison of serum antibody levels against each antigen between HDs and NF-pNET patients Scatter dot blots of antibody levels obtained by AlphaLISA^®^ with PTEN, EID3, EHD1, LGALS9, and BRAP recombinant GST-fusion proteins tested with sera from 25 NF-pNET patients and 25 HDs. Antibody levels against the EID3 antigen was significantly higher in the NF-pNET patient group than in the HD group (^*^p < 0.05, Mann-Whitney *U* test).

To compare the antibody reactivity against the five antigens in the HD and patient groups, we performed the AlphaLISA^®^ immunoassay using the sera of 25 HDs and 25 patients (Table 2). The results are shown in Figure 2. Among the five antigens, antibody levels against the EID3 antigen in the patient group were significantly higher than those in the HD group (7.034 ± 3.390 vs. 3.477 ± 2.289, *p =* 0.0005, Figure 2). There was no significant difference in antibody levels against the remaining four antigens (PTEN, EHD1, LGALS9, BRAP) between groups (Figure 2). These results demonstrate a correlation between serum-EID3 antibodies (anti-EID3 Abs) and the presence of NF-pNETs.

**Table 2:** Clinical features of NF-pNET patients and healthy donors in this study

		NF-pNET patients (*n* = 25)	HD (*n* = 25)
Gender	Male	9	9
	Female	16	16
Age: mean ± SD (years)		54.2 ± 10.6	53.5 ± 9.9
Tumor size: mean ± SD (mm)		26.7 ± 24.0	-
Grade	1/2	8/17	-
T	1/2/3/4	12/8/1/4	-
N	0/1	16/9	-
M	0/1	19/6	-
Stage	I/II/III/IV	10/4/5/6	-

HD: healthy donors

NF-pNET: non-functional pancreatic neuroendocrine tumor

### Correlation between serum anti-EID3 Ab levels and serum CgA levels

**Figure 3:**
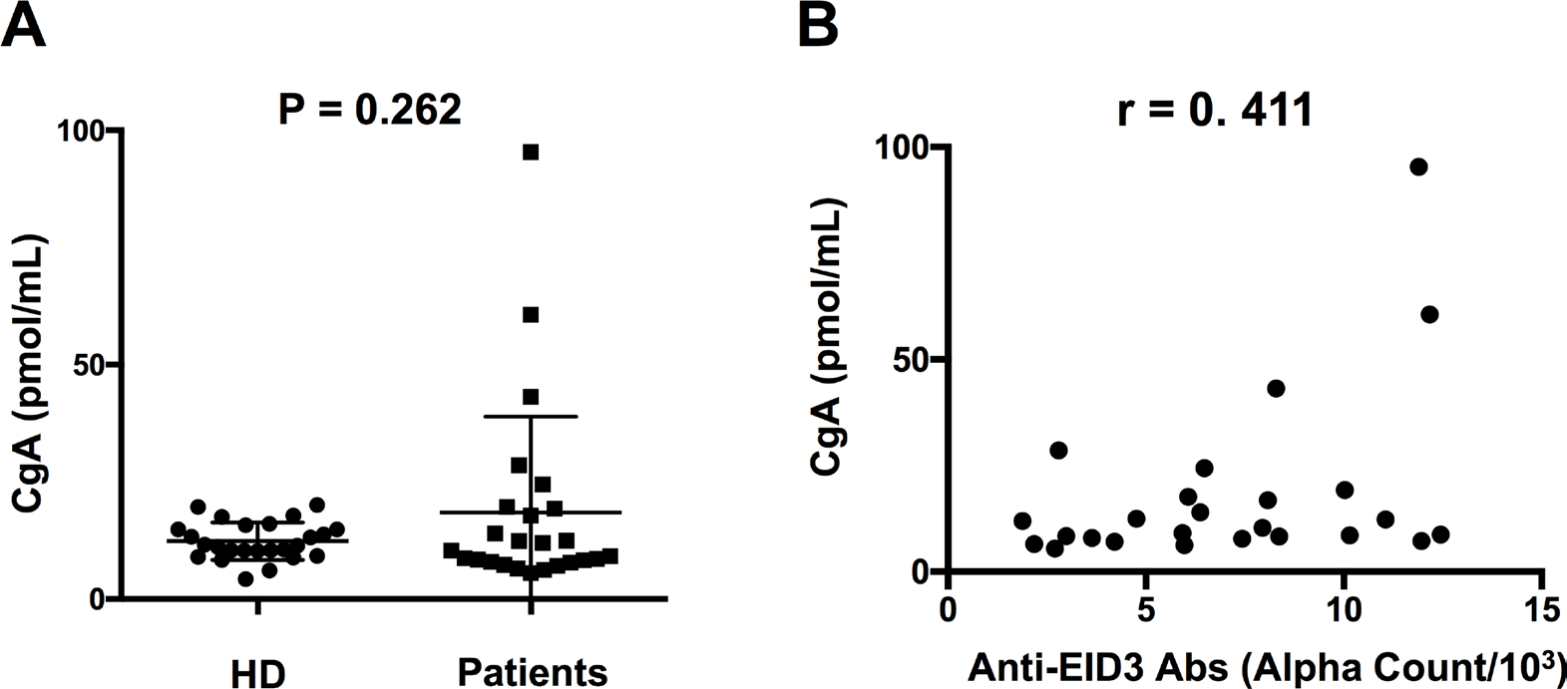
Correlations between serum CgA levels and anti-EID3 Ab levels in NF-pNET patients (**A**) Scatter dot blots of serum CgA levels tested with sera from 25 NF-pNET patients and 25 HDs. There was no significant difference in serum CgA levels between the two groups. (**B**) Correlation analysis of serum CgA and anti-EID3 Ab levels in patient group (*n* = 25). The serum levels of anti-EID3 Abs correlate with serum CgA levels. (*r* = 0.411, *p* = 0.041, Pearson’s correlation). CgA: Chromogranin A, NF-pNET: non-functional pancreas neuroendocrine tumor.

Serum CgA levels were measured in 25 NF-pNET patients and HDs. The results are shown in Figure 3A. The median values of CgA levels in NF-pNET patients and HDs were 11.93 (5.403–95.37) pmol/mL and 11.71 (range 4.487–22.12) pmol/mL, respectively. There was no statistically significant difference between the patient group and HD group. The correlation between serum anti-EID3 Ab levels and serum CgA levels are shown in Figure 3B. There was a relatively strong correlation between serum anti-EID3 Ab levels and serum CgA levels (*r* = 0.411, *p* = 0.041, Pearson’s correlation).

### Diagnostic accuracy of Anti-EID3 Ab and CgA levels in detecting NF-pNETs by ROC curve analysis

**Figure 4:**
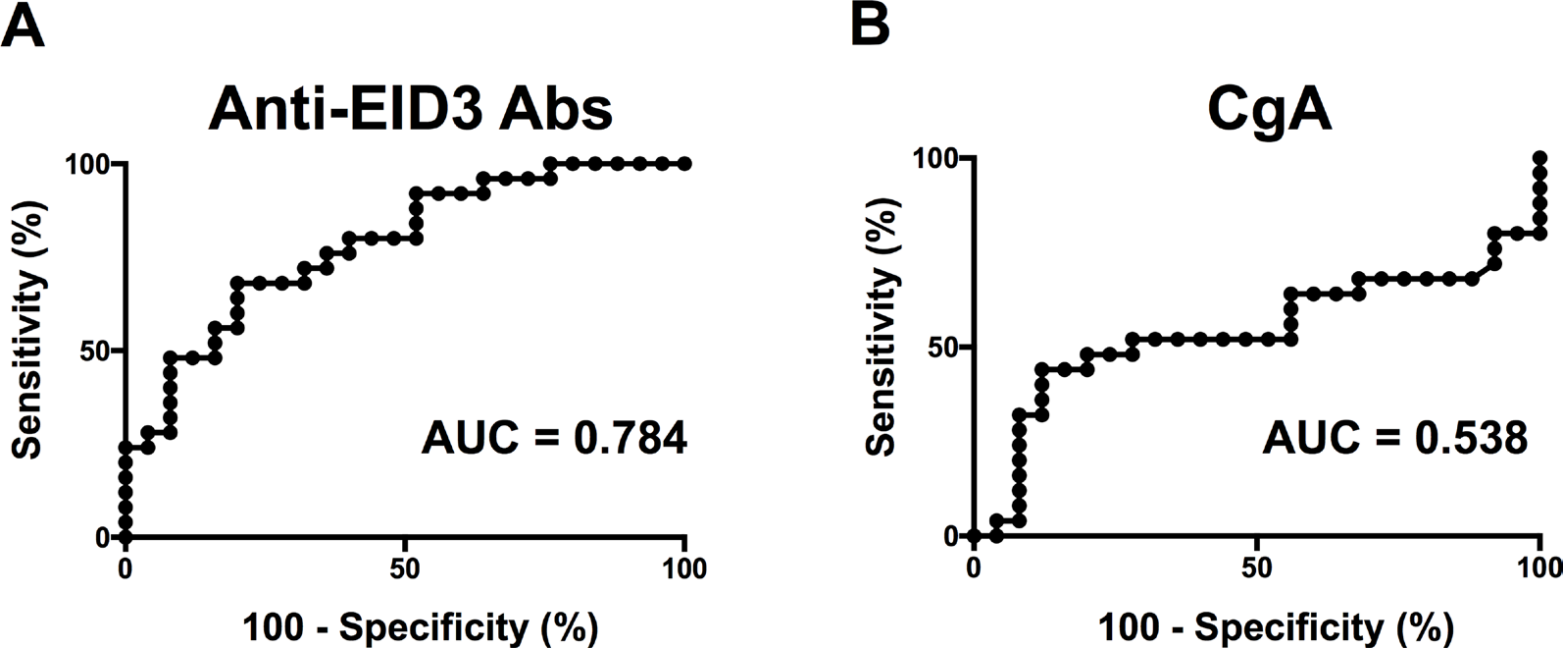
Diagnostic accuracy of Anti-EID3 Abs and CgA in NF-pNETs In order to evaluate the diagnostic potential of serum anti-EID 3 Ab and CgA in the detecting NF-pNET, ROC curve analysis was performed. (**A**) ROC curve analysis of serum anti-EID3 Ab levels in diagnosing NF-pNET patients. The AUC calculated from the results of the ROC curve analysis was 0.784 (95% CI: 0.658–0.909). (**B**) ROC curve analysis of serum Chromogranin A in diagnosing NF-pNET patients. The AUC calculated from the results of the ROC curve analysis was 0.538 (95% CI: 0.367–0.709). ROC: receiver operating characteristic, AUC: area under curve, NF-pNETs: non-functional pancreas neuroendocrine tumors.

We examined the accuracy of serum anti-EID3 Ab levels and serum CgA levels to serve as a tumor marker for NF-pNETs. ROC curves were drawn and the AUC was calculated to evaluate the diagnostic potential of serum anti-EID3 Abs and serum CgA. For serum anti-EID3 Abs, the AUC was 0.784 (95% confidence interval [CI]: 0.658–0.909) (Figure 4A). The sensitivity and specificity were 40% (95% CI: 0.211–0.613) and 92% (95% CI: 0.739–0.990), respectively, when the cut-off value of serum anti-EID3 Ab levels was set as the mean + two standard deviations (SDs) of the HD group (cut-off value: 8.055). For serum CgA, the AUC was 0.538 (95% CI: 0.367–0.709) (Figure 4B). The sensitivity and specificity were 32% (95% CI: 0.465–0.850) and 88% (95% CI: 0.593–0.932), respectively, when the cut-off value of serum CgA was set as the mean + two SDs of the HD group (cut-off value: 17.61). Then, combination assays were performed using serum CgA and anti-EID3 Ab levels results. Parallel combined testing was considered positive when one of the two assays showed a value above the cut-off value. The results showed that the sensitivity increased to 52%.

### Association between clinicopathological features and serum anti-EID3 Ab level

The patient group was divided into two groups according to the above cut-off value: EID3 Ab-positive, serum anti-EID3 Ab level ≥8.055; EID3 Ab-negative, serum anti-EID3 Ab level <8.055. Then we examined the relationship between clinicopathological features of NF-pNET patients and anti-EID3 Ab-positive classification (Table 3). There was no statistically significant association between anti-EID3 Ab positive rate and clinicopathological features.

**Table 3:** Association between serum anti-EID3 Abs positive rate and the clinicopathological features of NF-pNET patients

Characteristic		Cases	anti-EID3 Abs positive rate	*p* value
		*n*	*n* (%)	
Gender	Male	9	3 (33)	0.6913
	Female	16	7 (44)	
Age	<54	12	6 (50)	0.4283
	≥54	13	4 (31)	
Tumor size	<17 mm	12	5 (41)	>0.9999
	≥17 mm	13	5 (38)	
Grade	G1	8	4 (50)	0.6668
	G2	17	6 (35)	
Ki-67/MIB-1	<3%	11	6 (54)	0.2406
	3–20%	14	4 (28)	
T	T1, T2	20	6 (30)	0.1206
	T3, T4	5	4 (80)	
N	N0	16	5 (31)	0.3973
	N1	9	5 (55)	
M	M0	19	7 (37)	0.6532
	M1	6	3 (50)	
Stage	I, II	14	4 (29)	0.2406
	III, IV	11	6 (54)	

NF-pNET: non-functional pancreatic neuroendocrine tumor, Abs: antibodies

**Figure 5:**
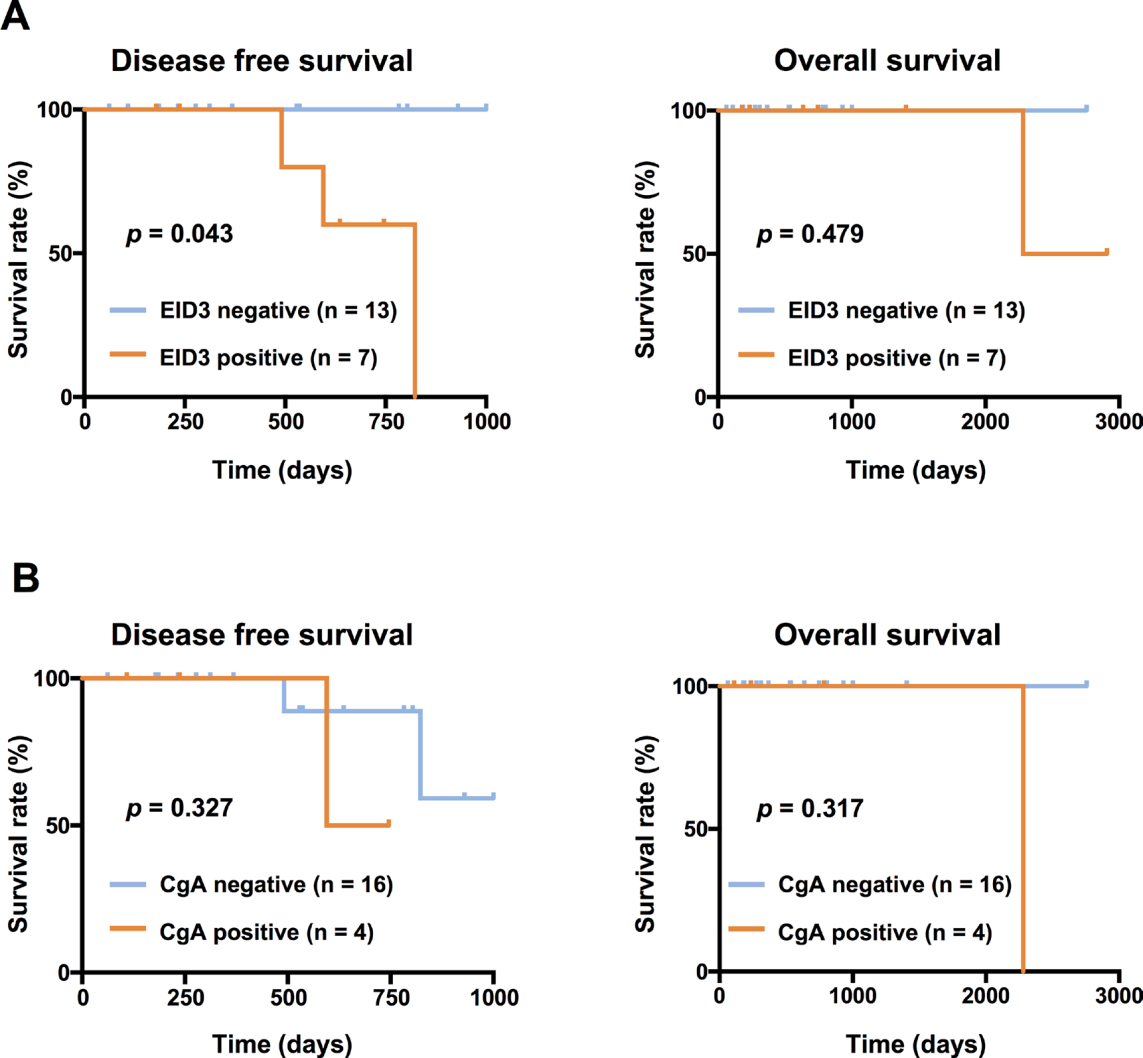
Kaplan-Meier survival curves depicting DFS and OS according to the level of serum anti-EID3 Ab and CgA levels The Kaplan-Meier plots show the relationship between serum anti-EID3 Ab and CgA levels and prognosis. The patient group was divided into two groups according to the cut-off value of serum anti-EID3 Ab levels set as the mean + two SDs of the HD group (EID3 Ab-positive: serum anti-EID3 Ab levels ≥ cut-off value, EID3 Ab-negative: serum anti-EID3 Ab levels < cut-off value). Patients who were serum anti-EID3 Ab-positive had a significantly shorter DFS than those who were Ab-negative (Log rank test, *p =* 0.0436). There was no significant correlation between serum anti-EID3 Ab level and OS. Similarly, the patient group was divided into two groups (CgA-positive: serum CgA levels ≥ cut-off value, CgA-negative: serum CgA levels < cut-off value, cut-off value: 17.61 pmol/mL). There was no significant difference between serum CgA levels and OS or DFS. DFS: disease free survival, OS: Overall survival, NF-pNETs: non-functional pancreas neuroendocrine tumors.

In addition, we performed survival analysis of 20 patients who underwent R0 resection. Univariate analysis showed that the EID3 Ab-positive group had significantly shorter disease free survival (DFS) than the EID3 Ab-negative group ( *p =* 0.043, Figure 5A). There was no statistically significant difference between serum CgA levels and OS or DFS (Figure 5B). Regarding other factors, primary tumor size and invasion to other organs (T) ( *p* = 0.006), regional lymph node metastasis (N) ( *p* = 0.020), and stage ( *p* = 0.020) were correlated with DFS; however, gender, age, grade, tumor size, distant metastasis (M), and overall survival (OS) did not correlate with DFS (all *p* values > 0.05) (Table 4).

**Table 4:** Univariate analysis of disease free survival analysis for NF-pNET patients

Patient characteristics	(Number of patient)	Hazard ratio	95% CI	*p*-value
All patients (20)				
Gender	Male (8) vs. Female (12)	3.793	0.3071 to 46.83	0.256
Age	≥54 (10) vs. <54 (10)	9.499	0.0104 to 1.060	0.056
Grade	G2 (13) vs. G1 (7)	2.369	0.2353 to 23.86	0.464
Tumor size	≥17 mm (10) vs. <17 mm (10)	10.15	0.9559 to 107.9	0.054
T	T3,4 (3) vs. T1,2 (17)	33.98	2.7520 to 419.6	0.006^*^
N	N1 (7) vs. N0 (13)	16.48	1.5520 to 175.1	0.020^*^
M	M1 (2) vs. M0 (18)	2.969	0.1413 to 62.39	0.483
Stage	III/IV (7) vs. I/II (13)	16.48	1.5520 to 175.1	0.020^*^
anti-EID3 Abs	positive (7) vs. negative (13)	10.78	1.0700 to 108.5	0.043^*^

NF-pNET: non-functional pancreatic neuroendocrine tumor, CI: confidence interval, Abs: antibodies

^*^p < 0.05

### Gene and protein expression of EID3 in NF-pNET and normal pancreatic tissues

**Figure 6:**
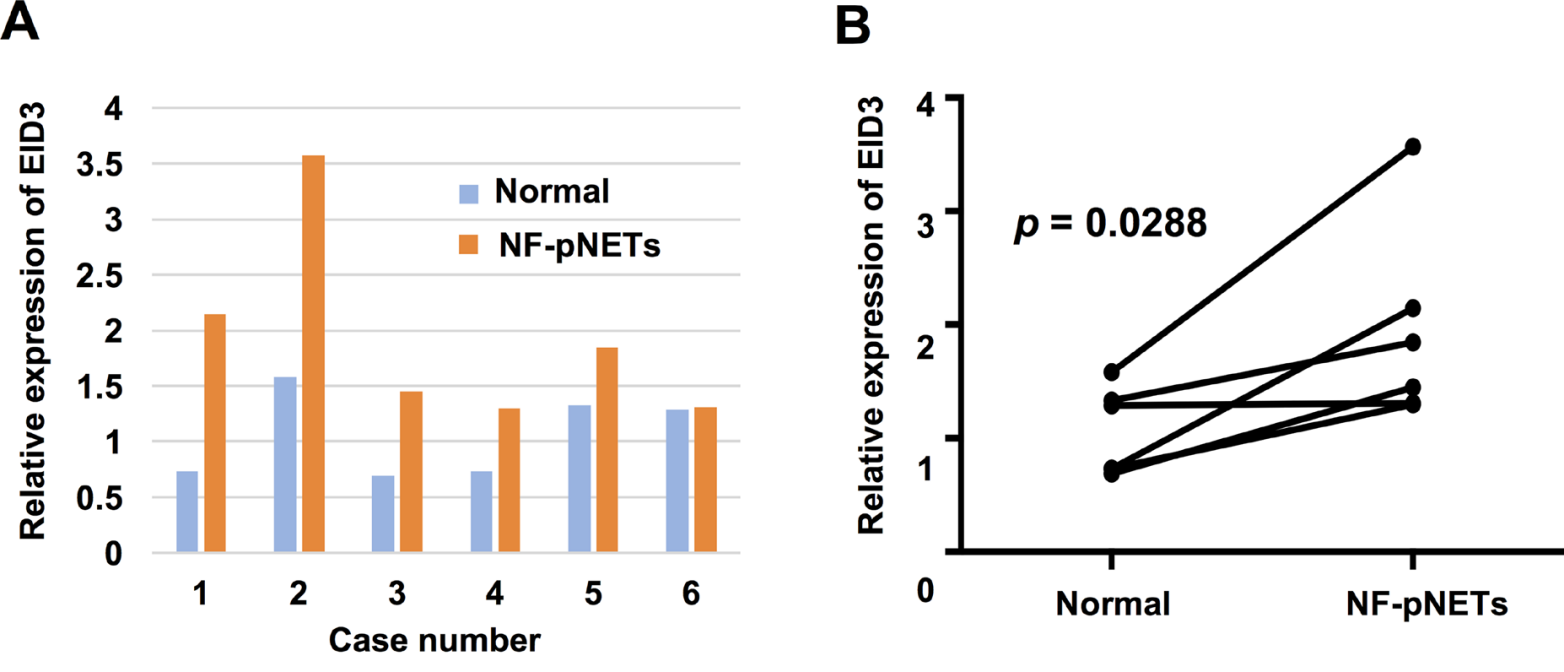
EID3 mRNA expression in NF-pNET and normal pancreatic tissue mRNA detection by qPCR in the NF-pNET and normal pancreatic tissues of six cases (**A**). Relative expression of EID3 was analyzed by the comparative CT method normalized by the housekeeping gene GAPDH. (**B**) The expression of EID3 was significantly higher in NF-pNET tissue than in normal pancreatic tissue (Paired *t*-test, *p* = 0.0288).

**Figure 7:**
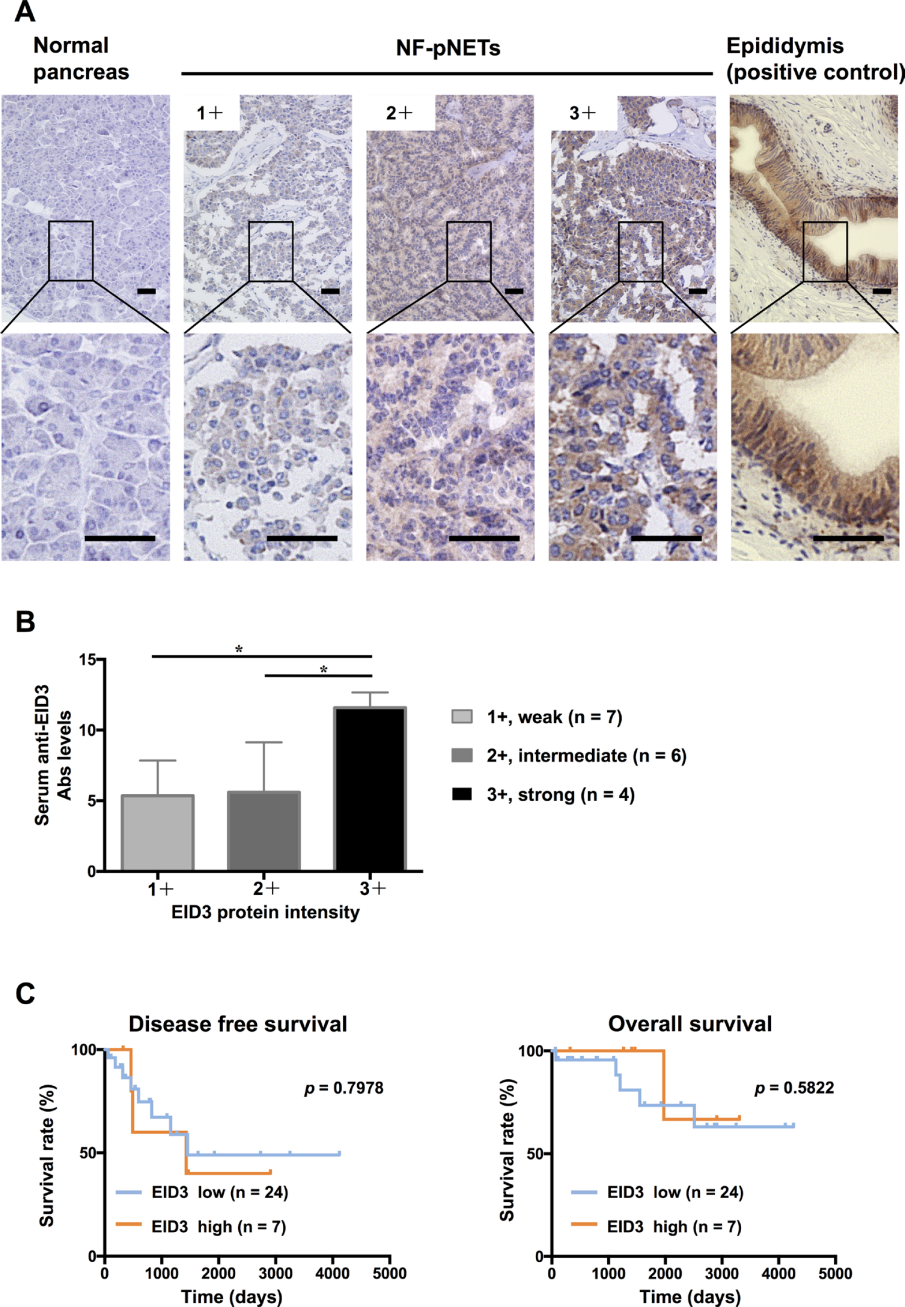
Analysis of EID3 protein expression in NF-pNETs and analysis of correlation between expression and prognosis (**A**) Endogenous EID3 protein expression was detected by immunohistochemical staining in normal pancreatic and NF-pNET tissues. Normal epididymis was stained as the positive control. Strong cytoplasmic expression of the EID3 protein was found in the NF-pNET compared to normal pancreas tissues. Depending on the case, weak (1+), intermediate (2+), or strong (3+) expression was observed compared to the epididymis. (**B**) Correlation between EID3 protein expression and serum anti-EID3 Ab levels. Serum anti-EID3 Ab levels were significantly higher in the strong (3+) EID3 protein expression group (^*^p < 0.05). (**C**) The Kaplan-Meier plots show the relationship between EID3 protein expression and prognosis. The patient group was divided into two groups based on the intensity of EID3 protein expression: low EID3 expression group (1+, 2+) and high EID3 expression group (3+). There was no significant correlation between protein expression and DFS and OS. Scale bar, 50 μm. DFS: disease free survival, OS: overall survival, NF-pNETs: non-functional pancreas neuroendocrine tumors.

Differences in the mRNA expression of EID3 in tumor and normal pancreatic tissues were analyzed by quantitative real time RT-PCR (qPCR) for six NF-pNET patients in which both tumor and normal pancreatic tissue were preserved (Figure 6A). There was higher expression of EID3 in NF-pNETs than in normal pancreatic tissue in five of the six cases (paired *t*-test, *p* = 0.0288, Figure 6B). The expression and distribution of EID3 proteins in tumor and normal pancreatic tissue was examined by immunohistochemical staining using 31 resected tissue samples from NF-pNET patients. Representative results (Figure 7A) showed that the NF-pNET tissue was more strongly stained than normal pancreatic tissue. Regarding the localization of the EID3 protein, it was more abundant in the cytoplasm than in the nucleus. There was no heterogeneity in the protein expression of EID3 in the tumor tissue. Depending on the case, weak (1+, Figure 7A), intermediate (2+, Figure 7A), and strong (3+, Figure 7A) expression was observed compared to the epididymis, which served as the positive control. In addition, the high EID3 protein expression was associated with high serum anti-EID3 Ab levels ( *p* < 0.05, Figure 7B) among the 17 patients whose tissue sample and serum were preserved. Next, we divided the patients into two groups based on the intensity of EID3 protein expression: low EID3 group (1+, 2+) and high EID3 group (3+). We examined the relationship among the clinicopathological features of the patients between the two groups, and found no significant association between EID3 protein expression and the clinicopathological features (Table 5). Finally, we performed survival analysis between the two groups, and found no statistically significant correlation between EID3 protein expression and DFS or OS (Figure 7C).

**Table 5:** Association with protein expression of EID3 in IHC and the clinicopathological features of NF-pNET patients

Characteristic		EID3 high (*n* = 7)	EID3 low (*n* = 24)	*p*-value
		*n*	*n*	
Gender	Male	2	11	0.6672
	Female	5	13	
Age	<54	4	12	>0.9999
	≥54	3	12	
Tumor size	<17 mm	3	9	>0.9999
	≥17 mm	4	15	
Grade	G1	1	10	0.3717
	G2	6	14	
T	T1, T2	3	11	>0.9999
	T3, T4	4	13	
N	N0	4	16	0.6757
	N1	3	8	
M	M0	6	20	>0.9999
	M1	1	4	
Stage	I, II	3	15	0.413
	III, IV	4	9	

NF-pNET: non-functional pancreatic neuroendocrine tumors, Abs: antibodies

## DISCUSSION

The purpose of this study was to identify novel serum antibodies in patients with NF-pNETs using SEREX screening, with patient sera as the detection platform. Five tumor antigens were identified, of which antibody levels against the EID3 antigen was significantly higher in the patient group. In addition, the AUC value calculated by ROC analysis indicated that serum anti-EID3 Abs may be a tumor marker with moderate accuracy.

EID3 was first cloned as the third member of the EID family; it inhibits cellular differentiation by binding to CREB-binding protein/p300 and suppressing transcription [18]. EID3 is highly expressed in the testis and is localized in both the nucleus and cytoplasm [19]. Under normal conditions, the EID protein is rapidly degraded in the proteasome through ubiquitin-dependent proteolysis by binding to the retinoblastoma tumor suppressor protein (pRB) at the end of the cell cycle [20, 21]. However, inactivating pRB mutations cause EID stabilization, resulting in inhibition of cell differentiation, which may be the mechanism by which EID family members contribute to tumorigenesis [18, 19, 22]. In fact, it has been reported that EID3 is involved in cell differentiation inhibition and cancer stem cell formation in colorectal cancer [23]. Regarding pNETs, pRB is reportedly inactivated through phosphorylation due to the high expression of Cdk4 [24], and such pRB inactivation may cause an increase in EID expression. Furthermore, phosphorylated pRB fails to suppress E2F, causing transcriptional activation of various factors related to cell cycle. The promoter region of the EID gene contains the E2F binding region (http://www.genecards.org/), and activation of E2F may lead to an increase in EID mRNA expression levels. In our study, the results of qPCR and immunohistochemical staining showed that EID3 mRNA and protein expression was higher in NF-pNETs than in normal pancreatic tissue. These results support the above mentioned mechanism that high EID3 expression may occur in pNETs.

The immune system usually produces antibodies in response to foreign proteins other than self antigens. When the immune system cannot distinguish between self- and non-self proteins, autoantibodies are produced. Although the mechanism underlying autoantibody production is not completely understood, it has been reported that it could be due to a genetic predisposition combined with an environmental trigger, such as a viral illness or prolonged exposure to certain toxic chemicals, or the overexpression of a normal antigen [25, 26]. For example, the p53 antibody has been discovered in the sera of animals with tumors induced by SV40, which stabilize wild-type p53 through its interaction with SV40 large T antigen [27, 28]. In this study, high EID3 expression was observed in NF-pNETs as determined by qPCR and immunohistochemical staining. Moreover, serum anti-EID3 Ab levels were significantly higher in the high EID3 protein expression group. These results suggest that high EID3 expression in tumors may cause antibody production. We also found that serum anti-EID3 Ab level, but not EID protein expression, correlated with DFS. This discrepancy may be due to the fact that Abs were also produced against small amounts of antigen, and even weak protein expression detected by immunohistochemical staining could lead to a high serum antibody level in the AlphaLISA^®^ immunoassay. Furthermore, immunohistochemical staining and AlphaLISA^®^ are different protein detection methods with different sensitivities. [29, 30].

Tumor markers can be divided into three groups based on clinical utility: diagnosis, prognosis, and predictive. Diagnostic markers are used to aid in diagnosis and recurrence screening; typical examples include prostate-specific antigen in prostate cancer and alpha-fetoprotein in liver cancer [31, 32]. Prognostic markers predict the course of a disease; for example, cancer antigen19-9 (CA19-9) in pancreatic cancer and carcinoembryonic antigen in colon cancer [32, 33]. Predictive biomarkers predict the response to chemotherapy and the probability of cancer recurrence; typical examples include BRCA1 in breast cancer and K-ras in colon cancer [34, 35]. These tumor makers have multiple roles; for example, in pancreatic cancer, CA19-9 is used as both a diagnostic and prognostic marker. In this study, we investigated the utility of serum EID3 Abs as a diagnostic and prognostic biomarker. To this end, antibody levels between the HD and NF-pNET groups were compared using AlphaLISA^®^, and the sensitivity and specificity were calculated when the cut-off value set as the mean + two SDs of the HD group. The results showed that the sensitivity and specificity of the anti-EID3 Abs were 40% and 92%, respectively, and the diagnostic accuracy was similar to that previously reported for CgA in the diagnosis of pNETs [36–38]. These data suggest that serum anti-EID3 Abs may be useful as a diagnostic marker for NF-pNETs. Furthermore, a combination assay with serum anti-EID3 Ab and CgA level improved the sensitivity to 52%. Thus, combination assays with serum tumor markers may be a useful approach for detecting NF-pNETs.

The most studied tumor marker for pNETs is CgA; however, it has the disadvantage of frequently causing false positives due to the presence of multiple secretory processes that cause increases in its concentration [36, 39–41]. In addition, CgA is not suitable for early detection because it is likely to be positive in an advanced disease stage [39, 42]. In contrast to CgA, the IgG-type tumor marker identified by SEREX screening is suitable for detection in the early stage because a relatively small amount of antigen causes a large immune response that increases the antibody concentration in the serum [12, 25, 26, 43, 44]. These favorable characteristics make serum anti-EID3 Abs a suitable candidate for use in the early detection and recurrence screening of NF-pNETs.

It has been reported that genetic abnormalities and expression by immunostaining of KRAS, DAXX/ATRX and MEN1 are associated with prognosis. As a representative example, gene mutations in DAXX/ATRX were observed in 53% of pNET patients and correlated with poor prognosis [40]. In addition, the low expression of DAXX/ATRX has also been reported to be a poor prognostic factor for pNET patients [45, 46]. To determine if EID3 correlated with pNET prognosis, the relationship between EID3 protein expression and prognosis, and the relationship between serum EID3 Ab levels and prognosis were evaluated. The immunohistochemistry results showed that EID3 protein expression was not associated with OS and DFS, but higher serum anti-EID3 Ab levels correlated with shorter DFS. Thus, serum anti-EID3 Ab levels may be useful as a predictive biomarker to detect the recurrence of NF-pNETs. The discrepancy between DFS and OS was mainly due to the fact that treatment outcomes of patients with pNETs have been significantly improved by multidisciplinary treatments including surgery and molecular targeted therapy even after recurrence. Therefore, there were only a few cases of death within the observation period due to the relatively good prognosis of most patients. This also applies to other breast and colon cancer malignancies (Prognoscan: http://www.abren.net/PrognoScan/, Munakata K *et al*. [23]). Thus, in terms of the contribution of EID3 to tumorigenesis via RB-related signaling pathways, high EID3 Ab levels alone do not appear to directly associate with shorter OS. Further verification experiments are needed. In the case of the anti-p53 antibody, which is a similar antibody-type tumor marker, the level of serum anti-p53 antibody was associated with poor prognosis in patients with colorectal cancer [47]. In that report, the authors tested pre-operative sera from 255 patients with colorectal cancer with an enzyme-linked immunoassay and the presence of p53 antibodies correlated with the following prognostic factors: histological differentiation grade, tumor invasion into blood vessels, shorter survival, and shorter disease-free survival. The presence of serum antibodies against antigens involved in such tumorigenesis may reflect genetic mutations and antigen overexpression, abnormalities that may enhance tumor progression [48].

There were several limitations in this study. First, because this was an exploratory study of a rare disease, only a small number of patients were enrolled. Furthermore, because of the small number of cases, multivariate analysis was not applicable in the survival analysis. Thus, to confirm these findings, additional large-scale studies are necessary. In addition, because nonspecific reactions were observed in the AlphaLISA^®^ immunoassay using GST-fusion proteins, which have epitopes other than target-specific epitopes leading to decreased sensitivity and specificity, further validations are warranted to improve sensitivity and specificity. This may be achieved by identifying epitopes for AlphaLISA^®^ detection utilizing shorter synthetic peptides specific to EID3, as previously reported [49]. This may also eliminate the possibility that anti-EID3 Ab will bind to other EID family members. We could not compare the serum anti-EID3 Ab levels before and after resection, because we have no consecutive pre- and post-resection sample. As a result of our CgA analysis, sensitivity was lower than previously reported results [38]. This is probably due to the fact that there were many cases of small tumor size and few cases of liver metastasis.

In conclusion, we identified five NF-pNET tumor antigens by the SEREX technology, of which the antibody levels against EID3 antigen were significantly higher in NF-pNET patients than in the HDs. Furthermore, the higher sera anti-EID3 Ab levels correlated to a shorter DFS in NF-pNET patients who underwent R0 surgery. These results suggest that serum anti-EID3 Abs may be useful as a diagnostic and predictive tumor marker.

## MATERIALS AND METHODS

### Human samples

To construct the cDNA libraries, the following NF-pNET tissues were obtained from three patients who underwent surgery at our department in 2016: NF-pNETs, Grade 1, T1N0M0 Stage I, no metastasis; NF-pNETs, Grade 2, T1N0M0 Stage I, no metastasis; and NF-pNETs, Grade 2, T3N1M1 Stage IV, liver metastasis. Sera were obtained from 25 patients with NF-pNETs who were diagnosed from 2009 to 2016 in our affiliated institution. Patients with other synchronous cancer and autoimmune diseases were excluded. Patients were classified according to the tumor-node-metastasis (TNM) classification of the European Neuroendocrine Tumor Society [50]. The Grade of tumor differentiation was classified based on the 2010 World Health Organization grading system [51]. A total of 20 of the 25 patients received R0 radical surgery, 3 of whom had liver metastasis during follow-up. Then 5 inoperable patients of the 25 patients were treated with chemotherapy, molecular-targeted drugs, a somatostatin analog, and transcatheter arterial chemoembolization as appropriate. All of the recurrent cases had liver metastasis. Twenty-five HDs matched for sex and age were used as normal controls (Table 1). For immunohistochemical staining analysis, 31 resected tissue samples were obtained from NF-pNET patients at our institute between 2003 and 2016. Tumor tissues and serum were stored at –80°C until use. The study protocol was approved by the Institutional Review Board of Hokkaido University Hospital (No. 015–0182).

### Preparation of λZAP II cDNA libraries

Total RNA was extracted from tumor tissues using the TRIzol^®^ Reagent (Thermo Fisher Scientific, Waltham, MA, USA) and mRNA was purified using the Oligotex™-dT30 <Super> mRNA Purification Kit (Takara Bio, Kusatsu, Japan). cDNA library was constructed from the mRNA with the cDNA Library Construction Kit (Takara Bio) and then ligated into the EcoRI-XhoI site of λZAP II vector after double digestion with EcoRI and XhoI (Lambda ZAP II Undigested Vector Kit, Gigapack III Gold packaging extracts: Agilent Technologies, Santa Clara, CA, USA). Additionally, human testis cDNA library (Uni-ZAP XR Premade Library) was obtained from Agilent Technologies.

### Screening of cDNA libraries by SEREX

The cDNA libraries were screened as previously reported [7]. *Escherichia coli* (*E. coli*) XL1-Blue MRF’ were infected with phage containing the cDNA libraries and then cultured on an NZY agar plate for 4 to 5 h until plaques began to appear. A nitrocellulose membrane immersed in IPTG for 30 min was placed on the NZY agar plate and cultured for 2 h, so that the expressed proteins would transfer from the cDNA clones to the membranes. After washing with TBS-T (0.05% Tween-20, 150 mM NaCl, 20 mM Tris-HCl [pH 7.5]), the membranes were blocked in 1% BSA for 1 h. Then the membranes were incubated for 1 h with a 1:2000 dilution of patient sera, which served as the source of the antibodies. After washing the membrane with TBS-T, the membranes were incubated with a 1:5000 dilution of peroxidase-conjugated affiniPure goat anti-human IgG (Jackson ImmunoResearch Laboratories, West Grove, PA, USA). After incubation with alkaline phosphatase color development solution (0.3 mg/ml nitro blue tetrazolium, 0.15 mg/ml 5-bromo-4-chloro-3-indolylphosphate, 100 mM NaCl, 5 mM MgCl_2_, 100 mM Tris-HCl [pH 9.5]), the positive plaques were visualized and picked up. The positive plaques were screened twice after which the clones were monoclonalized.

### Identification of genes encoding antigens by sequence analysis

The monoclonal phages containing cloned cDNA were converted to pBluescript phagemid by *in vivo* excision using the ExAssist helper phage with SOLR strain. The plasmid DNA was purified using GenElute Plasmid Miniprep Kit (Sigma-Aldrich, St. Louis, MO, USA). The plasmid DNA was sequenced by ABI PRISM 3130-Avant Genetic Analyzer and compared with the National Center for Biotechnology Information database using BLAST to determine the gene sequence.

### Construction of GST-fusion protein of each identified antigen

Recombinant GST-fusion proteins were constructed by different methods depending on whether the EcoRI and XhoI sequences were present in the sequence of the identified antigen. If they were not present, the plasmid DNA was double digested with EcoRI and XhoI enzymes. The digested cDNA fragments were electrophoresed on an agarose gel and purified using GeneElute™ Minus EtBr SPIN COLUMNS (Sigma-Aldrich). Then the purified cDNA fragments were ligated into the pGEX vector double-digested with EcoRI and XhoI using the DNA Ligation Kit <Mighty Mix> (Takara). On the other hand, if the EcoRI and XhoI sequences were present in the sequence of the identified antigen, the inserted cDNA fragments were ligated into pGEX vector with the In-Fusion HD Cloning system (Takara Bio). Then the pGEX vector was transformed into ECOS™ Competent *E. Coli* BL21 (NIPPON GENE, Tokyo, Japan). Positive transformants were selected on LB plates containing 50 μg/ml ampicillin and incubated at 37°C. The plasmid DNA was purified and the appropriate recombinants were confirmed by DNA sequencing. Expression of the GST-fusion proteins was induced by incubating the transformed *E. Coli* BL21 with 0.5 mM IPTG for 4 h at 25°C. The GST-fusion proteins were purified over the GSTrap FF column (GE Healthcare, Chicago, IL, USA) and the buffer was exchanged with PBS using the Amicon Ultra 15 filter (Merck Millipore, Billerica, MA, USA).

### SDS-PAGE and western blotting

The recombinant GST fusion proteins were dissolved in SDS buffer and boiled at 100°C for 3 min, after which its purification was confirmed by SDS-PAGE followed by Coomassie blue staining. To detect GST-tagged recombinant proteins, western blotting was performed using anti-PTEN (Origene Technologies, Rockville, MD, USA), anti-EID3 (Abcam, Cambridge, UK), anti-EHD1 (Abcam), anti-BRAP (Abnova, Taipei, Taiwan), anti-LGALS9 (GeneTex, Los Angeles, CA, USA), and anti-GST (Rockland, Gilbertsville, PA, USA) antibodies (Supplementary Table 1).

### Analysis by AlphaLISA^®^

Serum antibody levels of candidate antigens between the patient group and HD group were compared using the AlphaLISA^®^ immunoassay. Briefly, 2.5 μl of serum diluted 1:100 with AlphaLISA^®^ ImmunoAssay Buffer (Perkins Elmer, Waltham, MA, USA) and 2.5 μl GST or GST-fusion protein (10 μg/mL) was placed in a 384-well microtiter plates (Perkin Elmer). The mixture was incubated for at least 3 h at room temperature. Then, anti-human IgG Acceptor Beads (2.5 μl of 40 μg/ml) and Glutathione Donor Beads (2.5 μl of 40 μg/ml) were added, followed by a 14-day incubation. The plate was read using EnSpire Alpha microplate reader (Perkin Elmer). Serum antibody levels against the GST-fusion proteins were determined by subtracting the alpha counts for GST protein from the alpha counts for the GST-fusion proteins.

### Measurements of serum CgA levels

Serum CgA levels in NF-pNET patients (*n* = 25) and HDs (*n* = 25) were measured by ELISA utilizing the Human Chromogranin A EIA Kit (Yanaihara Institute Inc., Fujinomiya, Japan) according to the manufacturer’s protocol. All samples were tested in duplicate.

### qPCR

Total RNA was extracted from six cases of tumor and normal pancreatic tissue stored in PAXgene^**®**^ Tissue (Qiagen, Hilden, Germany) according to the manufacturer’s protocol, and was used for cDNA synthesis (Prime Script RT Master Mix, Takara Bio). cDNA products were used to amplify target genes using Power SYBR Green Master Mix (Thermo Fisher Scientific). PCR reactions and data analysis were performed in a StepOne Real-time PCR system (Thermo Fisher Scientific), using the comparative CT method normalized by the housekeeping gene GAPDH. The primers used in this study were as follows: GAPDH (Forward: 5′-GAAGGTGAAGGTCGGAGTC -3′, Reverse: 5′- GAAGATGGTGATGGGATTTC-3′), EID3 (Forward: 5′-GCCGACGTAGACCCAAAGC-3′, Reverse: 5′-GTTAAGGAGTTGTTCGCCGAG-3′). Each PCR reaction was performed in triplicate.

### Immunohistochemistry

Tissue specimens were obtained from surgically resected specimens of NF-pNET patients. Paraffin-embedded specimens sliced to 5 μm were deparaffinized and heated in a pH 9.0 solution at 95°C for 20 min using an antigen activation processor (Dako, Santa Clara, CA, USA) to activate the antigen. Endogenous peroxidase activity was blocked with Peroxidase-Blocking Reagent (Dako). Non-specific binding was reduced with 10% normal goat serum (Nichirei, Tokyo, Japan) and Antibody Diluent with Background Reducing Components (DAKO). The primary antibody was allowed to react overnight at 4°C. Following visualization step using EnVision FLEX/HRP system (Dako), the slides were stained with hematoxylin and covered with a cover slip.

### Statistical analysis

All of the statistical analyses were performed using GraphPad Prism 6 software (GraphPad Software Inc, La Jolla, CA, USA). To compare the different groups, Pearson Chi-square test, Fisher’s exact test, Paired *t*-test or Mann-Whitney *U* test was used as appropriate. Receiver operating characteristic (ROC) analysis was used to evaluate the diagnostic value of serum antibodies. Survival analysis was performed according to the Kaplan–Meier method in 20 patients underwent R0 radical surgery. The difference in survival rate was estimated by log rank test. The hazard ratio was calculated by the Cochran-Mantel-Haenszel method. A *p* value less than 0.05 was considered statistically significant. Statistical analysis was performed utilizing Pearson’s and Spearman’s rank correlation (95% CI) for continuous and ordinal variables, respectively.

## SUPPLEMENTARY MATERIALS TABLE



## Abbreviations

Abs: antibodies
AlphaLISA: amplified luminescent proximity homogeneous immunosorbent assay
AUC: area under curve
BLAST: Basic Local Alignment Search Tool
CgA: Chromogranin A
CI: confidence interval
DFS: disease free survival
ENETS: European Neuroendocrine Tumor Society
GST: glutathione S-transferase
HD: healthy donors
NCBI: National Center for Biotechnology Information
OS: overall survival
pNETs: pancreatic neuroendocrine tumors
pRB: retinoblastoma protein
ROC: receiver operating characteristic
RT-PCR: reverse transcriptase-polymerase chain reaction
SEREX: serological identification of antigens by recombinant cDNA expression cloning
TACE: transcatheter arterial chemoembolization
WHO: World Health Organization
