# Supplementary material for: Identification of novel serum autoantibodies against EID3 in non-functional pancreatic neuroendocrine tumors
Source: Oncotarget. 2017 Oct 31;8(63):106206–21. doi: 10.18632/oncotarget.22175 (PMC5739727; doi:10.18632/oncotarget.22175)
Supplement: Supplementary file 1 [file oncotarget-08-106206-s001.pdf]

Identification of novel serum autoantibodies against EID3 in non-functional pancreatic neuroendocrine tumors

SUPPLEMENTARY MATERIALS

Supplementary Table 1: List of antibodies used for immunohistochemical staining and Western blotting

**Immunohistochemical staining**			
Molecule	Clonality	Dilution	Manufacture
EID3	Rabbit polyclonal	1:1000	Abcam
**Western blotting**			
Molecule	Clonality	Dilution	Manufacture
PTEN	Mouse monoclonal	1:2000	Origene
EID3	Rabbit polyclonal	1:1000	Abcam
EHD1	Rabbit monoclonal	1:1000	Abcam
LGALS9	Rabbit polyclonal	1:1000	GeneTex
BRAP	Rabbit polyclonal	1:250	Abnova
